# Suicide Attempt in Patients With Gambling Disorder—Associations With Comorbidity Including Substance Use Disorders

**DOI:** 10.3389/fpsyt.2020.593533

**Published:** 2020-11-16

**Authors:** Anders Håkansson, Anna Karlsson

**Affiliations:** ^1^Department of Clinical Sciences Lund, Psychiatry, Faculty of Medicine, Lund University, Lund, Sweden; ^2^Malmö Addiction Center, Region Skåne, Malmö, Sweden

**Keywords:** gambling, suicide attempt, behavioral addiction, substance use disorder, comorbidity, gambling disorder

## Abstract

**Background:** Gambling disorder is known to be associated with increased risk of suicidal behavior. However, relatively little is known about how the risk of suicide attempts in gambling disorder is influenced by comorbid alcohol or drug use disorders, as well as other psychiatric conditions.

**Methods:** The present study is a nationwide, diagnostic register study assessing the risk of suicide attempts (including fatal ones) in gambling disorder in Sweden in 2005–2016.

**Results:** In a total of 2,099 individuals (23 percent women) with gambling disorder, 417 individuals had a suicide attempt (including 10 fatal cases of suicide) during the study period. Suicidal behavior was more common in patients with substance use disorders at any time during the study period (50 percent if both alcohol and drug use disorders were present, and 10 percent if none of these were present). In logistic regression, suicidal behavior was significantly associated with female gender (OR 2.13 [1.63–2.78]), mood disorders (OR 2.65 [2.00–3.50]), anxiety disorders (OR 1.78 [1.34–2.35]), and with alcohol (OR 1.95 [1.51–2.51]) or drug use disorders (OR 3.60 [2.76–4.69]), respectively.

**Conclusions:** Suicidal behavior in clinical gambling disorder patients is common, but markedly more common in the presence of substance use and other comorbid disorders.

## Background

Gambling disorder is a psychiatric disorder recognized as one of the addictive diseases in the most recent version of the diagnostic manual of the American Psychiatric Association (1). In recent years, an increasing amount of research has described the high level of psychiatric comorbidity in gambling disorder (2–4), including the increased risk of completed suicide in patients diagnosed with a gambling disorder (5). Likewise, in clinical treatment-seeking patients, suicidal ideation has been described to be highly prevalent (6). Also, gambling disorder was statistically associated with attempted suicide in a large sample of veterans (7), and in the general population, problem gambling has been seen to be associated with both suicidal ideation and attempted suicide (8). The risk of having committed a suicide attempt has been shown to be comparably high compared to patients with substance use disorders (9), and suicide attempts in the general population remain statistically associated with a gambling disorder even when controlling for substance use disorders (10). In total, it has been reported that somewhere between five percent and 40 percent of problem gamblers in any kind of treatment- or help-seeking contact report a history of a suicide attempt (11). Meanwhile, clinical assessment and treatment of a problematic gambling behavior may be problematic, as treatment seeking has been described to be low (12).

A substantial comorbidity between gambling disorder and substance use disorders (SUD) has been documented (3, 4, 13), and there are reasons to believe that a more complicated course in gambling disorder may be seen in case of a comorbid SUD (13). Given the well-established suicide risk in alcohol and drug use disorders, and the high comorbidity between gambling and SUD (3, 4, 13), it can be hypothesized that alcohol and drug use may be associated with suicidal behavior in patients with a gambling disorder. Still, these potential associations are hitherto only partly outlined. A study in a relatively limited sample of problem gamblers found that drug use was associated with a previous history of suicide attempts, and substance use was a factor separating problem gamblers who reported a suicide attempt from those reporting suicidal ideation but no attempt (14). Penfold et al. studied a small sample of 12 problem gamblers with a suicide attempt, among whom a majority also had alcohol problems (15). Also, Potenza et al. reported that in subjects calling a gambling telephone helpline, individuals also reporting alcohol problems were more likely to report a history of suicide attempts (16). Further, in some research assessing suicidal behavior in problem gamblers, alcohol and drug use either have not been studied at the same time (17), or, as in a Spanish clinical study of gambling disorder patients, scores of alcohol and drug problems did not differ significantly between patients with or without a history of suicide attempts (18). In a study comparing suicide attempts among treatment seeking patients with gambling or substance use disorders, comparable rates of suicide attempts were seen in the groups, although few study participants were diagnosed with both a gambling disorder and a substance use disorder (9). Also, in a study in a subsample of helpline callers with suicidal ideation, those who also reported a suicide attempt did not report significantly more alcohol or drug problems, although differences in absolute numbers were seen such that the study may be limited by its sample size (19). Thus, conclusions are hitherto difficult to draw regarding the potential role of substance use disorders in the risk of suicidal behavior in problem gamblers.

Thus, the present study, in a nationwide national register dataset of patients with a gambling disorder at some time, aimed to study whether the occurrence of suicide attempts would demonstrate associations with alcohol or drug use disorders, also when controlling for other common psychiatric disorder.

## Methods

The present study is based on the Swedish nationwide patient register, held by the Swedish Board of Health and Welfare. The Swedish patient register includes health care data for in-patient treatment and for all specialized out-patient treatment (i.e., not including primary care, “GP” visits). The same authority holds a cause of death register, where all deceased individuals in Sweden are registered, including both underlying and all contributing causes of death.

The present study includes all individuals who were registered with a gambling disorder (ICD-10 pathological gambling, F63.0) diagnosis at some time during 2005–2016. Previous reports of psychiatric comorbidity and associations with suicide death have been published from the same database (4, 5).

Clients were included only if an individual was at least 18 years of age at the first diagnosis of gambling disorder, thereby excluding 73 individuals. As in previous publications (4, 5), minors were excluded in order to eliminate the theoretical uncertainty about whether minors had truly been diagnosed with a disorder related to gambling for money, as the wording of “gambling disorder” in Swedish would not fully exclude a problem rather related to problem gaming.

The outcome measure of the present study was defined as a suicide attempt (diagnostic codes X60-X84 in the patient register at any time), or death from suicide (diagnostic codes X60-X84 in the cause of death register at any time).

The overall study of in-patient and out-patient register data for patients with gambling disorder was approved by the Regional Ethics Board, Lund, Sweden (file number 2016/1104).

### Statistical Methods

The occurrence of any suicide attempt during the study period was reported in descriptive analyses, including the reporting of these prevalence figures in patients with no alcohol or drug use disorder during the study period, with alcohol but no drug use disorder, with drug but no alcohol use disorder, or both. In an unadjusted analysis, patients with any suicide attempt during the study period were compared to patients without suicide attempts, with respect to age group at baseline, gender, and the occurrence of any alcohol use disorder, drug use disorder, psychotic disorders, mood disorders, and anxiety disorder, all compared with the chi-square test (categorical yes/no for most variables and linear-by-linear for age groups). Second, all variables were included in a logistic regression analysis, with any suicide attempt (yes/no) as the dichotomous dependent variable, and with each of the covariates above as independent variables. Data were reported as odds ratios with 95 percent confidence intervals. All analyses were carried out using the IBM SPSS statistical software, version 25.0 (20).

## Results

In total, 2,099 individuals were included, among whom 474 (23 percent) were women. Fifty-five percent of patients (*n* = 1,147) were ever hospitalized for a psychiatric disorder, during the study period. Nineteen percent (*n* = 407) had a suicide attempt, and 417 had either a suicide attempt or deceased due to suicide during the study period. During the study period, 29 percent (*n* = 618) had an alcohol use disorder, and 23 percent (*n* = 484) had a drug use disorder. Alcohol use disorders (30 percent of men and 29 percent of women, *p* = 0.68) and drug use disorders (23 percent of men and 25 percent of women, *p* = 0.41) were not associated with gender. Age groups differed between men and women, with women being significantly older at the start of the study period. Twenty-eight and 15 percent of men and women were below 20 years of age at study start, respectively, whereas nine and six percent, respectively, were 50 years or older (*p* < 0.001).

In patients with both an alcohol use disorder and a drug use disorder at some time during the study period (*n* = 270), 50 percent had a suicide attempt at some time during the study period. In patients with an alcohol use disorder (and no drug use disorder, *n* = 348), the prevalence was 20 percent, and in patients with a drug use disorder (but no alcohol use disorder, *n* = 214) the prevalence was 36 percent. Among patients without any alcohol or drug use disorder during the study period (*n* = 1,267), 10 percent had a suicide attempt at some time.

In unadjusted analyses, having a suicide attempt or suicide death was significantly associated with female gender, as well as with all the diagnostic groups assessed (psychotic disorders, mood disorders, anxiety disorders, alcohol use disorders, and drug use disorders, all *p* < 0.001), as well as with higher age group (*p* = 0.03, linear-by-linear) as can be seen in Table 1. In logistic regression analysis, including all these diagnostic categories along with age group and gender, suicidal behavior remained significantly associated with female gender (OR 2.13 [1.63–2.78]), alcohol use disorders (OR 1.95 [1.51–2.51]), drug use disorders (OR 3.60 [2.76–4.69]), mood disorders (OR 2.65 [2.00–3.50]), and anxiety disorders (OR 1.78 [1.34–2.35]), but not with age group or psychotic disorders as can be seen in Table 2.

**Table 1:** Comparison of gambling disorder with or without any suicide attempt/suicide during the study period (*N* = 2,099).

	**Yes (*n* = 417), % (*n*)**	**No (*n* = 1,682), % (*n*)**	***p*-value**
**Age group**	22 (90)	26 (445)	0.03^*^
1–19			
20–29	30 (126)	29 (496)	
30–39	22 (93)	23 (379)	
40–49	18 (75)	15 (252)	
50–59	6 (26)	5 (82)	
60–69	2 (7)	2 (28)	
Female gender	36 (150)	19 (324)	<0.001
Psychotic disorder	14 (59)	8 (137)	<0.001
Mood disorder	80 (333)	49 (819)	<0.001
Anxiety disorder	79 (330)	55 (922)	<0.001
Alcohol use disorder	50 (208)	24 (410)	<0.001
Drug use disorder	51 (213)	16 (271)	<0.001

*Age groups, gender distribution, and psychiatric comorbidity in patients with or without suicide attempt/suicide, respectively. Chi-square analyses*.

* *Linear-by-linear*.

**Table 2:** Variables associated with any suicide attempt/suicide during the study period (*N* = 2,099), all variables adjusted for one another.

	**OR**	**95% confidence interval**
Female gender	2.13	1.63–2.78
Age (per increasing age group)	1.06	0.96–1.17
Psychotic disorders	1.31	0.91–1.90
Mood disorders	2.65	2.00–3.50
Anxiety disorders	1.78	1.34–2.35
Alcohol use disorders	1.95	1.51–2.51
Drug use disorders	3.60	2.76–4.69

*Non-stepwise logistic regression analysis with any suicide attempt/suicide history as the dependent variable.Odds ratios and 95 percent confidence intervals*.

## Discussion

The present study demonstrated that attempted suicides in gambling disorder, including fatal attempts, are markedly more common in individuals who are also diagnosed with alcohol and drug use disorders, also when controlling for age and gender and for psychiatric diagnostic groups. Altogether, in a nationwide sample of individuals diagnosed with a gambling disorder in specialized health care settings, suicidal behavior is very common, and common even in the absence of a substance use disorder, but markedly more common for individuals for whom any substance use disorder is present over time.

The link between suicide attempts in patients with a gambling disorder and a comorbid alcohol- or drug-related diagnosis was consistent with the findings from a smaller sample by Hodgins et al. (14), and with the higher reporting of suicide attempts in those reporting comorbid alcohol problems among helpline callers (16).

The association of suicidal behavior with mood disorders and female gender was consistent with the study of Bischof et al., who reported an association between these variables and self-reported suicidal events in a sample of problems gamblers (21). Likewise, in a clinical sample where suicidal risk was estimated from the MINI diagnostic interview, this risk was seen to be associated with depressive and anxiety disorders (22). Although suicide attempts in patients with a gambling disorder were clearly more common in the context of any substance use disorder, it is reasonable to assume that in cases with both a gambling- and a substance-related disorder, both conditions may have had an influence on the suicidal process, and it is beyond the scope of the present study to analyze the role of each separate condition. However, Kausch described in 2003 that in a sample of patients with a gambling disorder, a large majority also had a substance use disorder, but even among them, a large percentage of patients with a suicide attempt reported their attempt to be gambling-related (23). Thus, this further points to the need to address problematic gambling behaviors in the assessment of suicidality in patients with poor mental health, and that even in the presence of another disorder known to increase suicide risk, gambling may be of interest to screen for and to diagnose at an early stage. Screening for gambling problems has been called for in mental health services, where a number of brief screening tools have been suggested, and where the prevalence of problem gambling is likely markedly higher than in the general population (24). Furthermore, active screening in specialized mental health services may be of particular importance given the association with suicidal behavior seen in the present study. Such screening routines may also be relevant and feasible to implement in primary care settings, which likely also see a substantial number of problem gamblers but for primarily other reasons than the gambling itself (25).

Importantly, the notion that individuals with gambling disorder and comorbid SUD suffer a more complex course is strengthened by our results indicating an increased presence of prior suicidal behavior in the group. Even heredity for SUD is common and may be associated with a more severe clinical picture in gamblers (26). However, in spite of the severity of the gambling and SUD comorbidity, few treatment studies are available for specific interventions in this comorbid group, apart from adapting evidence-based strategies for each condition separately (13). In addition, gambling disorder is a condition that can be treated, and where a range of support groups or evidence-based treatments are available. Most importantly, cognitive-behavioral therapy and motivational interventions are seen as efficacious (13, 27, 28). The findings of the present study, again underlining the gravity of gambling disorder and even more so in the presence of an alcohol or drug use disorder, call for implementation of such evidence-based treatments widely in society, and call for further research in groups with comorbidity and/or heredity for the combined conditions.

In addition to the high prevalence of psychiatric comorbidity in gambling disorder, including a high comorbidity with SUD, the associations between problem gambling and poor mental health may be bi-directional; i.e., whether the two conditions may occur in parallel or subsequently may by highly individual. Research from recent years confirms the picture of these associations going in different directions (29), but also, this would also be consistent with the well-established pathways model, describing three types of mechanisms underlying a gambling disorder in different patient groups, and where mental health problems are likely to differ (30). In the pathways model, problem gamblers are referred to as being either behaviorally conditioned, emotionally vulnerable, or antisocial/impulsivist gamblers. With respect to that paradigm, emotionally vulnerable gamblers have been described to be attracted to chance-based games providing a relief from emotional problems, rather than to skills-based games (31). Thus, from a standpoint of psychiatric comorbidity and risk of suicidal behavior, this may seem more likely to occur in emotionally vulnerable gamblers. While this goes beyond the scope of the present study, future studies addressing suicidal behavior in gambling disorder patients may need to deepen the understanding of the temporality and consecutive order of other mental health conditions in patients with a gambling problem.

The present study has implications for the risk assessment and prevention of suicidal acts in problem gamblers. Importantly, in the assessment of treatment-seeking patients with problem gambling, the findings of the present study support the active screening for substance-related disorders, given their close association to suicidal behavior. Also, it has been shown that debts are particularly associated with suicide in problem gambling (32), and mental health consequences of problem gambling need to be understood in the context of indebtedness (33). Also, among suicide victims whose suicides were judged to be committed in the context of a gambling disorder, gambling-related debt was common in a Hong Kong post-mortem study (34). Thus, beyond the context of health care professionals such as in mental health services, screening for problem gambling in consumer credit applicants has been suggested, in order to target a risk population in close association of a potentially gambling-related harm (35).

The present study has limitations. Although it included a nationwide, full-population sample of individuals receiving a gambling disorder diagnosis in the health care system, it is well-known that such registers do not cover a large percentage of the general population who fulfill criteria of a gambling disorder, both due to low treatment seeking, and due to the fact that many other institutions may provide support services outside of the medical system (36). Formal help seeking may be low and self-recovery in gambling disorder may be substantial (37). Likewise, primary care is not included in the national register used in the present study. Primary care has been suggested as a setting where problem gambling should be identified (38). This may be particularly important for the suicide-preventive aim of early problem gambling detection, as the conditions described here as correlates of suicidal behavior, such as mood disorders and substance use disorders, are likely to be diagnosed in primary care to a large extent. However, in the present setting, primary care is unlikely to have played a major role in gambling disorder diagnostics during the study period, as primary care has only been scientifically addressed as a potential arena for problem gambling screening, rather than hitherto being an active stakeholder in the clinical routine (25). This may decrease the significance of the study limitation related to the lack of primary care data in the national diagnostic registers.

In addition, data on prescribed and received pharmacological and psychological treatments were not available in the present study. A majority of psycho-social or psychological interventions are provided by social services which are not covered by national health care registers. Also, with respect to evidence-based pharmacological treatment in substance use disorders, their distribution in national registers are unlikely to cover a large share of the patients receiving SUD treatment (39), and a large share of SUD treatment is likely to have occurred outside of formal treatment systems (40). Likewise, structured treatment of gambling disorder would have provided an important additional co-variate in the present analyses, as several treatment methods are available and have provided evidence in recent years' research (28). However, structured treatment has been limited throughout the study period in the present setting, and also may have occurred either within social services or in the format of voluntary peer support provided by patient organizations (4). While adjustment for treatment data would have enriched the present data, thereby presenting a limitation to the present study, future studies should preferably combine broader measures of diagnostic and treatment data, making correlations with more socio-demographic characteristics possible.

In conclusion, the present study, in a nationwide register material of patients with a gambling disorder, demonstrates that the high prevalence of suicide attempts in gambling disorder patients is particularly high in individuals who also have another psychiatric disorder during the time studied here, and that alcohol and drug use disorders markedly increase the risk of suicidal behavior in gambling disorder patients. The findings call for improved screening and treatment interventions for patients with gambling disorder and other mental health comorbidity.

## Data Availability Statement

The datasets presented in this article are not readily available according to the decisions made by the ethics committee and the Swedish government authority responsible of nationwide register data. Requests to access the datasets should be directed to Anders Håkansson, anders_c.hakansson@med.lu.se.

## Ethics Statement

This study was reviewed and approved by the Regional Ethics Committee of Lund, Sweden (file number: 2016/1104). Written informed consent from the participants' legal guardian/next of kin was not required to participate in this study in accordance with the national legislation and the institutional requirements.

## Author Contributions

AH carried out the statistical analyses and wrote the first draft of the paper. All authors made substantial contributions to the extension and finish of the paper, are responsible of the overall research idea and the background data, and are equally responsible of the interpretation of data.

## Conflict of Interest

The authors declare that the research was conducted in the absence of any commercial or financial relationships that could be construed as a potential conflict of interest.
